# Evaluation of a Multivalent Vaccine against Lymphatic Filariasis in *Rhesus macaque* Model

**DOI:** 10.1371/journal.pone.0112982

**Published:** 2014-11-17

**Authors:** Gajalakshmi Dakshinamoorthy, Agneta von Gegerfelt, Hanne Andersen, Mark Lewis, Ramaswamy Kalyanasundaram

**Affiliations:** 1 Department of Biomedical Sciences, University of Illinois College of Medicine at Rockford, Rockford, Illinois, United States of America; 2 Bioqual Inc., Rockville, Maryland, United States of America; London School of Hygiene and Tropical Medicine, United Kingdom

## Abstract

Lymphatic filariasis affects 120 million people worldwide and another 1.2 billion people are at risk of acquiring the infection. Chemotherapy with mass drug administration is substantially reducing the incidence of the infection. Nevertheless, an effective vaccine is needed to prevent the infection and eradicate the disease. Previously we reported that a multivalent fusion protein vaccine (r*Bm*HAT) composed of small heat shock proteins 12.6 (HSP12.6), abundant larval transcript-2 (ALT-2) and large extracellular domain of tetraspanin (TSP LEL) could confer >95% protection against the challenge infection with *Brugia malayi* infective larvae (L3) in mouse and gerbil models. In this study we evaluated the immunogenicity and efficacy of r*Bm*HAT fusion protein vaccine in a rhesus macaque model. Our results show that r*Bm*HAT is highly immunogenic in rhesus macaques. All the vaccinated monkeys developed significant titers of antigen-specific IgG antibodies against each of the component antigens (16,000 for r*Bm*HSP12.6), (24,000 for r*Bm*ALT-2) and (16,000 for r*Bm*TSP-LEL). An *in vitro* antibody dependent cellular cytotoxicity (ADCC) assay performed using the sera samples from vaccinated monkeys showed that the anti-r*Bm*HAT antibodies are functional with 35% killing of *B. malayi* L3s. Vaccinated monkeys also had antigen responding cells in the peripheral blood. Vaccine-induced protection was determined after challenging the monkeys with 500 *B. malayi* L3. Following challenge infection, 3 out of 5 vaccinated macaques failed to develop the infection. These three protected macaques had high titers of IgG1 antibodies and their PBMC secreted significantly high levels of IFN-γ in response to the vaccine antigens. The two vaccinated macaques that picked the infection had slightly low titers of antibodies and their PBMC secreted high levels of IL-10. Based on these findings we conclude that the r*Bm*HAT vaccine is highly immunogenic and safe and can confer significant protection against challenge infections in rhesus macaques.

## Introduction

Human lymphatic filariasis caused mainly by *Wuchereria bancrofti* and *Brugia malayi* affects 120 million people and elicits a wide spectrum of pathological disorders of the lymphatic system with varied clinical manifestations. The filarial parasites can survive in the human for many years causing permanent disability due to chronic syndromes such as lymphoedema, elephantiasis, and hydrocoele. According to the World Health Organization, lymphatic filariasis is the second leading cause of physical disability in the world [1]. Certain individuals who live in the endemic regions remain retractile to filarial infection and carry high titer of circulating antibodies against select parasite antigens [2]. Previous studies showed that these serum antibodies are cytotoxic to infective larvae of the parasite (L3), suggesting that these individuals are naturally immune to the infection [3], [4]. These putatively immune individuals are called Endemic Normal (EN) [2]. Infected individuals do not carry these host protective antibodies [4]. Using an iterative screening of a phage cDNA expression library of the parasite with the sera from EN subjects, we identified several parasite antigens that were specifically recognized by the antibodies in the sera of EN subjects [5]. Subsequently, these antigens were cloned and their vaccine potential was evaluated in a mouse and/or jird models [3], [4], [6]–[8]. These studies identified three antigens [small heat shock proteins 12.6 (HSP12.6), abundant larval transcript-2 (ALT-2) and large extracellular domain of tetraspanin (TSP LEL)] as the most promising vaccine candidates. Vaccination trials with a multivalent fusion combination of the three proteins (r*Bm*HAT) showed that approximately 95% protection can be achieved against challenge infections with *B. malayi* L3 in a mouse and jird model of lymphatic filariasis [9]. Although mice and jirds do not develop the typical lymphatic pathology during lymphatic filariasis infection, they are an excellent model to dissect out some of the key preclinical parasitological and immunological changes. Since r*Bm*HAT vaccination gave close to sterile immunity in rodent models, there is great potential for developing this vaccine for human clinical trials. However, due to qualitative differences in the immune systems between rodents and human, vaccination trials in rodents are not easily translatable to clinical trial protocols. Also to understand the mechanisms of protective immune responses induced by vaccines, it is critical to use an animal model in which the immune system closely mimics that of human. Monkeys infected with *B. malayi* exhibited immunological changes in the lymphatic system similar to human lymphatic filariasis [10]. Therefore before testing the vaccine in human there is a need to evaluate its safety and immunogenicity in a non-human primate (NHP) model. Several other vaccines that are currently in use against infectious diseases were also developed through studies in NHPs [11], [12].

Rhesus macaques (*Macaca mulatta*) have close phylogenic relationship with human and are natural hosts for the lymphatic filarial infections [13]. In macaques filarial parasites develop into mature adult male and female worms and produce microfilaria similar to that in the human [14]. The infected macaques also develop fever, lymph node enlargement (around 5 weeks), lymphatic pathology (around 10–12 weeks) and chronic symptoms of elephantiasis very similar to that in the human lymphatic filariasis [15], [16]. Therefore, rhesus macaques are extremely valuable and an ideal animal model to evaluate the safety and efficacy of a vaccine against lymphatic filariasis [10], [17]. In fact, rhesus macaques have been used previously to test the vaccine potential of gamma-irradiation attenuated third stage *B. malayi* L3. In these previous studies, approximately 71% of vaccinated macaques were protected (5 out of 7 animals) against a challenge infection with *B. malayi* L3 [18] confirming that it is possible to evaluate vaccine-induced protection in macaques.

In the present study we evaluated the safety, immunogenicity and level of protection conferred following vaccination with r*Bm*HAT in a rhesus macaque model. Protection was determined by evaluating the titer of protective antibodies, cytokine response of antigen responding peripheral blood mononuclear cells (PBMC), lack of establishment of challenge infection (as determined by the presence of circulating microfilariae, Mf), and absence of any clinical signs of lymphatic filariasis.

## Materials and Methods

### Ethics statement

Use of Rhesus Macaques (*Macaca mulatta*) in these studies was approved by the IACUC (Animal ethics) committee at the Bioqual Inc. (Rockville, MD). Ten 3- to 5-year old male rhesus macaques were purchased from PrimGen (Hines, IL). All macaques were quarantined for 6–8 weeks before entering them into the studies. Macaques were paired for housing, blood and stool samples were analyzed to ensure that the macaques are negative for any infections. Before entering each macaque into the study, serum samples were analyzed to confirm that the animals do not carry circulating antigens or antibodies against *B. malayi* adult worm antigens.

Animals were housed at Bioqual’s facility in Rockville, MD. Care and husbandry were provided in compliance with federal laws and guidelines as well as in accordance with recommendations provided in the NIH guide and other accepted standards of laboratory animal care and use. Bioqual is accredited by the Association for the Assessment and Accreditation of Laboratory Animal Care, (AAALAC file #624) and holds an Assurance on file with the National Institute of Health, Office for Protection of Research Risks as required by the US Public Health Service Policy on Humane Care and Use of Laboratory Animals. The PHS Animal Welfare Assurance File Number is #A-3086-01.

Animal are cared for in rooms that are maintained at a temperature range of 68°F–74°F and humidity between 30% and 70%. The air handling equipment provides 10 to 15 filtered fresh (non-recirculated) air changes per hour for all animal rooms. All animal rooms are negative to the corridors as required for housing nonhuman primates and animals exposed to infectious agents. The exhaust is passed through a HEPA filter system on the roof (the air is not recirculated). The exhaust system HEPA filters are certified twice each year by an outside vendor to ensure satisfactory operation of the system. To accommodate socialization of animals, Bioqual has designed a 6.0 sq. ft. over/under system to accommodate two animals.

The animals are fed Primate Biscuit (15% Primate Diet 8714), supplied by Harlan Teklad, Madison WI. Fresh fruit are supplied by the L&M Produce Company. Food and water are provided daily to all monkeys. Daily diets are supplemented with fresh fruit, including apples, bananas, pears, and oranges.

Bioqual employs four fulltime veterinarians who oversee animal enrichment in accordance with Bioqual’s Behavioral Environment Enhancement Program (BEEP). Bioqual has worked with OLAW and clients to socially house as many animals as possible. Any animal that requires additional enrichment due to abnormal behavior has a behavior and enrichment plan tailored to its needs, which is supervised by the behaviorist in conjunction with Bioqual’s veterinarians, assigned technicians and animal caretakers. All NHPs are observed twice per day and evaluated bi-monthly by a fulltime employed behaviorist. Additional behavioral observations are performed as needed when recommended/requested by the veterinary staff. All NHPs receive at least three commercially available pet toys in their cages at all times to manipulate as part of their enriched environment. NHPs are given destructible enrichment as a way for them to alter their environment, release aggression/tension, and forage through.

The destructible enrichment provided includes items such as cardboard, shredded paper, and phonebooks. Soft toys, such as fleece blankets and soft pet toys, are available for comfort to all NHPs, but are given more frequently to young, sick, and/or behaviorally distressed individuals. Various types of feeders/grooming boards, such as corn feeders, turf feeders, paint rollers, fruit feeders, and coconuts may be given.

To control of pain and discomfort, animals were sedated with ketamine, sometimes in combination with xylazine or telazol, for all technical procedures. Ketamine were given intramuscularly in the amount necessary for short-term procedures such as blood drawing. Animals were fully anesthetized for the challenge procedure.

All methods of euthanasia are performed in accordance with the 2000 Report of the AVMA Panel on Euthanasia and 2007 AVMA guidelines on euthanasia. The euthanasia procedure is performed by intravenous injection of a sodium pentobarbital overdose and subsequent auscultation of the heart”.

Macaques were paired for housing, blood and stool samples were analyzed to ensure that the macaques are negative for any infections. Before entering each macaque into the study, serum samples were analyzed to confirm that the animals do not carry circulating antigens (Hha I PCR, please see below for details) or antibodies against *B. malayi* or *W. bancrofti* adult worm soluble antigens or against SXP-1 as determined by ELISA.

### Parasites

*B. malayi* infective third stage larvae (L3) were obtained from the NIAID/NIH Filariasis Research Reagent Resource Center (University of Georgia, Athens, GA).

### Multivalent fusion protein r*Bm*HAT

The multivalent fusion protein r*Bm*HAT expressed in *Escherichia coli* BL21 (pLysS), was purified and endotoxin removed by Pierce High Capacity Endotoxin removal resin column (Thermo Fisher Scientific, Rockford, IL) as described previously [9].

### Immunizations of r*Bm*HAT

Five macaques each received 200 µg of r*Bm*HAT vaccine mixed with 100 µg of AL007 alum (IDRI, Seattle, WA) under ABSL-2 conditions. Five (5) macaques that received alum (AL007) only remained as controls. Each animal was anesthetized with ketamine/xylazine and the vaccine was administered intramuscularly in each thigh (one injection site per thigh per vaccination). Animals were immunized at 4 weeks interval on days 0, 28 and 56. Intramuscular route is commonly used for clinical vaccine trials and hence we followed the same procedure for macaques. The injection sites were monitored daily for signs of fever, any adverse reactions (redness, swelling, etc.) for up to 7 days post immunization.

### *B. malayi* L3 challenge

On day 84, one month after the final dose of vaccine, macaques were anesthetized with ketamine HCl and challenged subcutaneously with 400–500 *Brugia malayi* L3. To facilitate the production of the relatively large number of L3 (500 L3/animal) required for challenging 10 immunized macaques, the animals were divided into 2 subgroups within each group. The subgroups were challenged one week apart. Before challenge *B. malayi* L3 were counted and examined for viability under a microscope. Only viable parasites were used for challenge.

### Monitoring of each animal after challenge

All animals were monitored daily for clinical signs after the challenge. Behavioral observations were similarly conducted during the entire post-challenge period. Clinical monitoring included serum chemistry, hematology, complete blood count (CBC) analysis (IDEXX) and CD4+/CD8+ T cell flow cytometry analysis. Body weights, body condition, lymphoedema and lymph node measurements were also recorded each time the animal was sedated for procedures (like immunizations, challenge, and blood collections).

### Sample collections

Blood samples and peripheral blood mononuclear cells (PBMC) were collected at the Bioqual primate facility and shipped to UIC for sample analyses. Whole blood was collected into BD Vacutainer SST tubes according to manufacturer’s instructions. Heparinized blood (1 ml) was collected from the femoral vein of each animal during the immunization period and from the saphenous vein during the challenge period. The shift in blood collection site was to eliminate any potential interference with the inguinal lymph node measurements or assessments of edema. Blood samples were obtained at multiple time points during the entire follow-up period.

### Isolation of PBMC

The blood pellets after plasma separation was diluted in phosphate buffered saline (PBS; 1∶2) and subjected to gradient density centrifugation for 30 min at 2200 rpm using a 90% Histopaque separation solution (Sigma, St. Louis, Mo.). The opaque interface containing mononuclear cells was collected, washed three times in PBS by centrifugation at 800 rpm. PBMC were enumerated using Trypan blue dye exclusion method and resuspended in RPMI 1640 medium containing 10% FBS (100 U/ml Penicillin/Streptomycin, and 2 mM L-glutamine). PBMC collected before the challenge was analyzed for T cell proliferation and IFN-γ secretions. PBMC collected after the challenge experiments were tested for T cell proliferation and ELISPOT assays. Proliferation assay was performed with PBMC isolated on the same day of blood collection. PBMC suspended in RPMI media with 10% FBS were used for Antibody Dependent Cellular Cytotoxicity (ADCC) assay and for cytokines analysis.

### T cells proliferation and flow cytometry

Carboxyfluorescein diacetate succinimidyl ester (CFSE) based assay was used for assessment of antigen-specific proliferation within the T cell population [19]. A 5 mM CFSE stock solution (Invitrogen, Grand Island, NY) was prepared according to manufacturer’s instructions. PBMC collected four weeks after the final immunization were gently resuspended at 10^7 ^cells/ml in 5 µM CFSE and incubated in the dark at 37°C for 15 minutes. Cells were centrifuged and washed with RPMI containing 10% FBS (100 U/ml Penicillin/Streptomycin, and 2 mM L-glutamine) and incubated for an additional 30 minutes at 37°C. Cells were then washed, resuspended in RPMI containing FBS, plated in a 24-well plate at 2×10^6^ cells/ml per well and incubated overnight at 37°C. The medium (∼500 µl) was removed the following day and cells were stimulated with 1 µg/mL of r*Bm*HAT. Samples incubated only with RPMI medium served as negative controls. As a positive control for each animal, cells were stimulated with phytohemagglutinin (PHA). Cells were cultured and harvested after 5 days of stimulation. Following a washing step with PBS/0.2% FBS, cells were surface stained with an antibody cocktail of CD3-APC-Cy, CD4-PE and CD8-PerCP and incubated for 20 minutes at room temperature. After an additional washing step with PBS/0.2% FBS the cells were acquired on BD FACS Canto II flow cytometer (BD, San Jose, CA) and analyzed on a BD FACS Diva Software v6.1.2. At least 50,000 events within the live lymphocyte gate were acquired.

### Cell Counts, serum chemistry and Complete Blood Count (CBC) analysis

CBC, serum chemistries and eosinophil counts were analyzed using commercial automated hematology and serum chemistry analyzers by IDEXX. Samples collected prior to the initiation of the study served as a normal reference baseline for each animal.

### Secreted levels of IFN-γ were measured using an ELISA

PBMC (1×10^6^ cells) were stimulated *in vitro* with 1 µg/ml of r*Bm*HAT for 5 days at 37°C. Following stimulation the supernatants were harvested and assayed for secreted levels of IFN-γ using an ELISA kit (Mabtech AB, Ashburn, VA) according to manufacturer’s instructions.

### ELISPOT assay

An ELISPOT assay was performed to determine the antigen-specific IFN-γ and IL-10 secreting cells in the PBMC of vaccinated and control macaques. We used a monkey ELISPOT kit purchased from U-Cytech biosciences (Yalelaan, The Netherlands) to determine the spot forming units as per the manufacturer’s instruction. PBMC collected 20 weeks post challenge were plated in 96 well plates at 1×10^6^ cells/ml and were stimulated with 100 ng/well of *B. malayi* adult soluble antigen (BmA) for 24 hours at 37°C and 5% CO_2_. Wells of ELISPOT plates were coated with 100 µl/well of capture antibodies (anti-IL-10 or anti- IFN-γ) diluted in sterile coating buffer and incubated overnight at 4°C. Plates were washed 2 times with sterile coating buffer. After blocking the plates with 200 µl/well of blocking buffer for 1 hour at room temperature, PBMC that were already stimulated with BmA antigens or only media (negative control) were added to the wells of the ELISPOT plates at 100 µl/well and incubated for 24 hrs at 37°C and 5% CO_2_. All the cells were removed from the plates and the membrane was washed 3 times with sterile PBS. Following wash, 100 µl of detection antibodies were added to each well and incubated at room temperature for 2 hr. After washing the plate 4 times with wash buffer, avidin-HRP reagent was added (100 µl/well) and incubated for 45 minutes at room temperature. After a final wash with PBS, freshly prepared 3-amino-9-ethylcarbazole (AEC) substrate solution was added (100 µl/well) and monitored for the development of spots at room temperature for 10–60 minutes. The substrate reaction was stopped by washing wells 3 times with 200 µl/well ultrapure water. The plates were air dried. Spots were counted using a dissecting microscope. The plates were stored in the dark prior to reading. Antigen-specific responses were determined by subtracting the number of spots in the negative control wells from the wells containing antigens. Results are shown as the mean value of spots obtained from triplicate wells.

### Analysis of serum antibody titers in macaques

Levels of IgG, IgG1, IgG2, IgG3, IgA and IgE antibodies against r*Bm*HSP, r*Bm*ALT-2, r*Bm*TSP or r*Bm*HAT were determined in the sera (collected one month after the final dose of vaccine) of each rhesus macaque using an indirect ELISA as described previously [9]. Briefly, wells of a 96 well microtiter ELISA plates were coated with 100 ng/well of antigens (r*Bm*HSP, r*Bm*ALT-2, r*Bm*TSP or r*Bm*HAT) in 0.05 M carbonate-bicarbonate buffer, pH 9.6. The wells were blocked with 3% BSA in 0.05% Phosphate Buffer Saline-Tween 20 (PBS-T), and 100 µl of sera samples (diluted in the range of 1∶100–1∶50,000 in PBS-T) from each macaque were added to each well. Goat anti-monkey IgG antibodies conjugated to peroxidase (Rockland Immunochemicals, Gilbertsville, PA) was used as secondary antibodies to determine IgG titer antibodies. The color was developed using OPD substrate and absorbance was read at 492 nm in the ELISA reader (BioRad, Hercules, CA). To determine the levels of isotype antibodies, biotinylated anti-monkey IgG1 (1∶2000), IgG2 (1∶200), IgG3 (1∶2000), IgA (1∶2000) and IgE (1∶1000) antibodies (NHP Reagent Resources, Boston, MA) were used as secondary antibodies. After washing the plates, optimally diluted streptavidin conjugated horse radish peroxidase (HRP) was added and further incubated for 60 minutes at room temperature and the color was developed.

### Antibody dependent cell mediated cytotoxicity assay (ADCC)

ADCC was performed according to the protocol described previously [9]. PBMC were prepared from heparinized whole blood from a naive healthy animal as described previously. Briefly, ten *B. malayi* L3 (suspended in 50 µl RPMI 1640 medium containing 10% FBS) were incubated with 2×10^5^ PBMC (in 50 µl RPMI 1640) and 50 µl of serum from each animal (collected one month after the final dose of vaccine) in a 96-well round bottom tissue culture plate. 5 replicates were performed for each serum sample. Control wells contained *B. malayi* L3 incubated in media, with sera alone or cells alone. The plates were incubated at 37°C with 5% CO_2_ for 48 hours. Following incubation, *B. malayi* L3 were examined under a microscope at 24 and 48 hours to determine larval viability. Dead L3 were defined as those having a limpid or straight appearance with no movements for an additional observation period of 8 hours at 37°C. Live larvae were active, coiled and motile. The percentage larval death was expressed as the ratio of the number of dead L3 to that of the total number recovered within the experimental period multiplied by 100. Average larval death in 5 wells were calculated and expressed as percent protection in each animal.

### Knott test to determine microfilaremia in macaques

The presence of Mf in the blood of macaques was detected using the Knott technique as described previously [20]. Peripheral blood of macaques was screened weekly for Mf starting from 5 weeks to 20 weeks post challenge. Briefly, whole blood was mixed with 9 ml of a 2% formalin solution (prepared in PBS) in a 15 ml conical centrifuge tube. The tubes were gently rocked for 2 min at room temperature and centrifuged at 1,500 rpm for 5 min. The supernatant was then thoroughly decanted by turning the tube completely upside down to remove all the liquid. Following this 5 ml of ACK lysis buffer (Quality Biologicals, Gaithersburg, MD) was added to the pellet and the tube was vortexed. Two to three drops of methylene blue solution (Fisher Scientific, Hannover Park, IL) was then added to the tubes, gently mixed, and smeared onto five glass slides. The samples were allowed to dry and read under a microscope using 40X lens objective. A comparison of Mf counts in blood collected from the saphenous and femoral veins showed similar results.

### Detection of Mf in the peripheral blood by polymerase chain reaction (PCR)

PCR-based assays are more sensitive in detecting the presence of Mf in the blood samples [21], [22]. Therefore, we used the PCR based assay also to confirm the presence of Mf in the blood samples of all macaques 20 weeks after challenge. Whole blood samples were centrifuged at 10,000 rpm for 5 min and the supernatant containing serum was stored at −20°C. DNA was isolated from the pellet using DNeasy Blood & Tissue Kit (Qiagen, Valencia, CA) according to the manufacturer’s instruction. Primers were synthesized at Integrated DNA Technologies Inc., (Coralville, IA) for HhaI tandem repeats. Primer sequence for HhaI tandem repeat was (Forward 5′-GCG CAT AAA TTC ATC AGC-3′, Reverse 5′-GCG CAA AAC TTA ATT ACA AAA GC-3′). PCR parameters were initial denaturation of 94°C for 5 min, followed by 40 cycles of 1 min at 94°C, 1 min at 56°C, 1 min at 72°C and a final extension of 10 min at 72°C. Following PCR reaction, 10 µl of each PCR product was analyzed on a 1% agarose gel.

### PBMC proliferations assay

PBMC collected 10 weeks post challenge were cultured in 96 well tissue culture plates at a concentration of 1×10^6^ cells/well in RPMI 1640 supplemented with 10% FCS. Cells were stimulated either with r*Bm*HAT antigen (1 mg/ml) or Concanavalin A (1 mg/ml) or with medium alone (unstimulated) in triplicate wells. PBMC were stimulated in triplicate wells and the plates were incubated at 37°C in 5% CO_2_. After 72 h, cell proliferation was measured using cell counting kit (CCK-8) (Dojindo Molecular Technologies, Inc, Gaithersburg). Stimulation index of PBMC proliferation was calculated using the formula: Absorbance of stimulated cells/Absorbance of unstimulated cells.

### Statistical analysis

Data are represented as the mean ± standard error. One-way ANOVA tests (Kruskal-Wallis) was performed for the antibody titer and T cell proliferation using GraphPad Prism software. Student T test was performed for protection studies. A probability (P) value of ≤0.001 was considered statistically significant.

## Results

### r*Bm*HAT vaccination did not induce any adverse reactions in macaques

The injection sites were monitored closely for signs of any adverse reactions (redness, swelling, etc.) for 7 days post immunization. There were no adverse reactions in any of the vaccinated or control animals. Clinical monitoring showed no dramatic loss of body weight (>10% of the original weight), changes in eating habits or any other behavioral changes. Temperature measurements obtained daily following immunizations did not show any significant variations. Temperature measurements were also performed at regular intervals using implanted transponders. There were no significant variations in the body temperature in vaccinated and control animals.

The lymph nodes in the left and right leg of all animals were monitored weekly starting approximately 2 weeks prior to challenge (to establish a baseline) and throughout the challenge period. The lymph nodes were measured with a caliper and observed for edema. The measurements showed an overall increase in the mean size of the inguinal lymph nodes in both legs during the 5–8 week post challenge period in all groups. Compared to the baseline (14.5 mm) the lymph node size in control animals were 22±1 mm and r*Bm*HAT group were 26.2±1 mm. Following this period, the sizes of the lymph nodes decreased to near pre-challenge levels in all macaques.

Challenge with *B. malayi* L3 did not alter the body temperature in macaques (data not shown). Analyses of the serum chemistry and hematology (CBC) values showed that they were all in the normal range for all cell types (data not shown) except for a slight increase in the eosinophil counts following L3 challenge in infected animals.

### All three antigens in the multivalent vaccine construct were immunogenic in macaques

**Figure 1:**
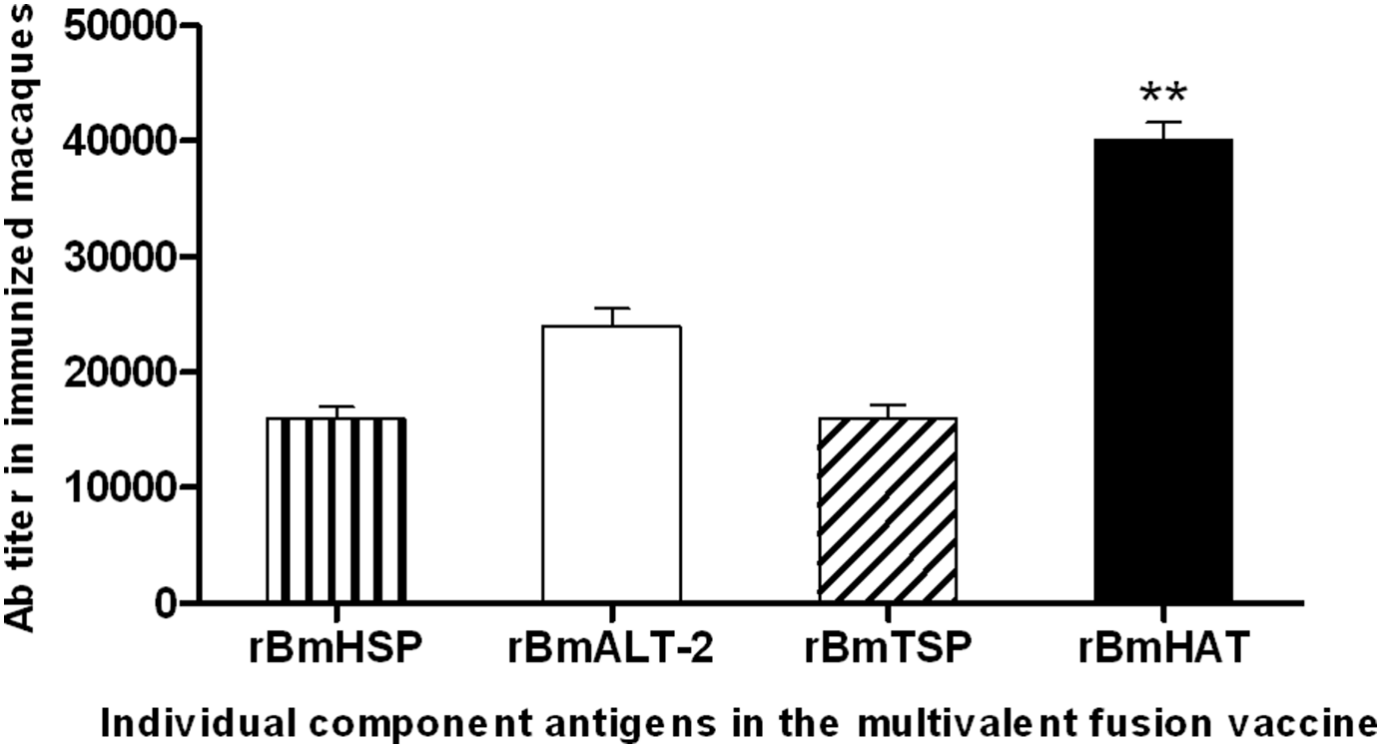
r*Bm*HAT specific IgG antibodies in the sera of macaques. Macaques were immunized three times at four weeks interval with 200 µg of r*Bm*HAT combined with alum (AL007) adjuvant. Sera collected one month after the final immunization was analyzed for IgG titer by ELISA. Approximately, 100 ng of each recombinant protein (100 ng/100 µl/well) were coated onto the wells of an ELISA plate and bound serum IgG was detected using a HRP labeled anti-monkey IgG secondary antibodies. Each bar represents mean ±S.D of 5 animals. Significant **P<0.001 titer of antibodies observed compared to other antigens.

Analysis of the IgG antibody titer in vaccinated macaques showed that all the macaques developed high titers (1∶40,000) of IgG antibodies after third immunization against r*Bm*HAT (Fig. 1). We then analyzed the titer of antibodies against each of the three component antigens in the vaccine construct. All macaques developed high titers of IgG antibodies against r*Bm*HSP12.6 (1∶16,000), r*Bm*ALT-2 (1∶24,000) and r*Bm*TSP-LEL (1∶16,000) (Fig. 1). There were slight individual variations in the titer of antibodies between each vaccinated macaques. On a comparative basis, macaque #5242, #5258 and #5259 showed the highest titer of IgG antibodies against the component antigens (except anti-r*Bm*HSP12.6 antibodies in macaque #5258 and anti-r*Bm*TSP antibodies in macaque #5259). Macaque #4996 and 5254 developed only low titers of antibodies to r*Bm*ALT-2 and r*Bm*TSP (Table 1).

**Table 1:** Titer of IgG antibodies.

	Animals immunized with r*Bm*HAT				
**Animal ID**	**4996**	**5242**	**5254**	**5258**	**5259**
**Antibody titre against r** ***Bm*** **HSP12.6**	6400	16,000*	6400	6400	16,000*
**Antibody titre against r** ***Bm*** **ALT-2**	3200	24000**	800	24000**	24000**
**Antibody titre against r** ***Bm*** **TSP**	16,000*	16,000*	12,800*	16,000*	6400
**Antibody titre against r** ***Bm*** **HAT**	40,000	40,000	40,000	40,000	40,000

Macaques were immunized with 200 µg of r*Bm*HAT with alum adjuvant. Anti- r*Bm*HAT antibodies against r*Bm*HSP12.6, r*Bm*ALT-2, r*Bm*TSP LEL or r*Bm*HAT were evaluated. Each animal differed in the antibody titer against each antigen.

*P<0.05 and **(*P*<0.001) statistically significant IgG antibody titer compared to other animals.

**Figure 2:**
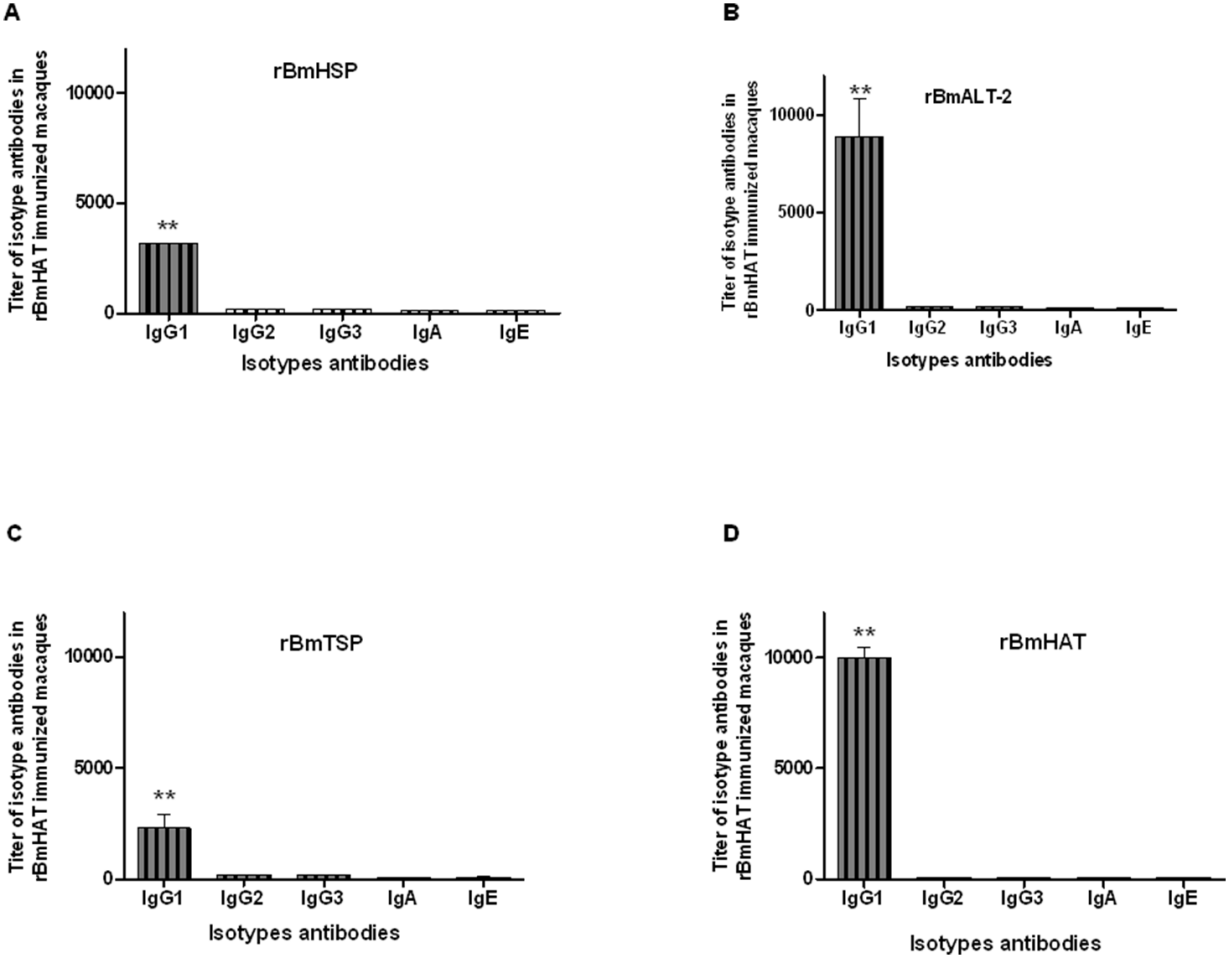
r*Bm*HAT specific antibodies isotypes in the sera of macaques. Levels of IgG1, IgG2, IgG3, IgA and IgE antibodies against A) r*Bm*HSP, B) r*Bm*ALT-2, C) r*Bm*TSP and D) r*Bm*HAT were determined in the sera (collected one month after the final dose of vaccine) of each rhesus macaque using an indirect ELISA. IgG1 antibodies were predominantly present in the immunized animals against all the four antigens tested. Each bar represents mean ±S.D of 5 animals. Significant **P<0.001 titer of antibodies observed compared to other animals.

Isotype analysis showed that nearly all of the antibodies were of IgG1 isotype against all the four antigens tested (r*Bm*HSP, r*Bm*ALT-2, r*Bm*TSP and r*Bm*HAT). Levels of IgG2, IgG3, IgA and IgE did not show any significant difference from the background values (Fig. 2).

### r*bm*hat responding cells were present in the pbmc of immunized rhesus macaques

To determine the antigen specific proliferative responses, PBMC was collected four weeks after the final vaccination. Cell proliferation was determined after stimulating CFSE labeled PBMC with r*Bm*HAT proteins for 5 days and counting the labeled cells in a flow cytometer. These results showed that the proliferation frequency of antigen-responding cells in the immunized animals were 3 folds higher (stimulation index 6.1±0.86) compared to the control animals (stimulation index 2.2±1.42). As expected, PBMC from all the animals showed robust proliferative responses (stimulation index 87.4±0) upon stimulation with pan-T mitogen, PHA. PBMC cultured in control medium had only low-level proliferation following 5-day incubation. The proliferation frequency value for each sample was obtained by subtracting the medium alone control value.

**Figure 3:**
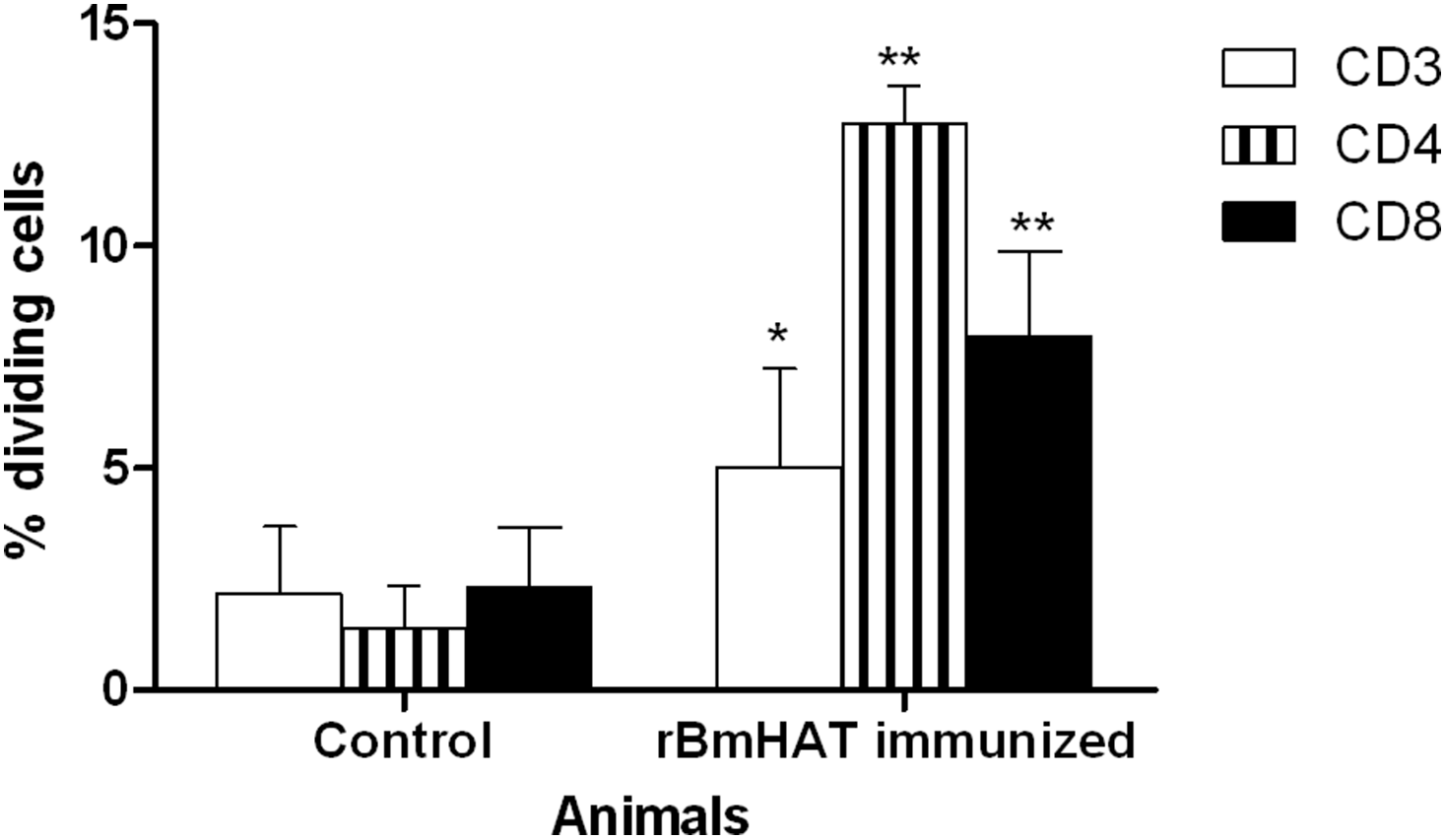
T cells proliferative responses in r*Bm*HAT vaccinated macaques. PBMC from immunized macaques isolated at 4 weeks post final immunization were stimulated with r*Bm*HAT or medium only (negative control). Flow cytometry analysis for the loss of CFSE labeling (indicating cell proliferation) was performed for cells within the live T cell (CD3+) or CD4+ or CD8+ T cell subsets. Shown are the mean ± S.I and results (% proliferating cells) of the five macaques in each group. The frequency for each sample is the value obtained following subtraction of the medium control. Significant **P<0.001 and *P<0.05 proliferation of cells observed compared to controls.

Frequency of CFSE labeled CD3+, CD4+ and CD8+ PBMC proliferating in response to antigen stimulation were determined by flow cytometry. These studies showed that there was an increase in the proliferation of antigen-responding T cells in all immunized macaques compared to control macaques (Fig. 3). Subset analysis showed that in immunized animals approximately 12.7% of the antigen responding T cells were CD4+ cells and 7.9% of T cells were CD8+ subsets (Fig. 3). Background proliferation in the presence of r*Bm*HAT antigen in the PBMC of control animals were 1.4% for CD4+ cells and 2.3% for CD8+ cells.

### Antigen responding cells in the PBMC of immunized monkeys secrete IFN-γ

Antigen responding cells in the spleen of r*Bm*HAT immunized mice and gerbils predominantly secreted high levels of IFN-γ [9]. Therefore, we wanted to determine if macaques also show a similar response after immunization but before challenge. These studies showed that PBMC from the 3 immunized macaques (#5242, #5258 and #5259) all secreted significant amounts of IFN-γ when stimulated with r*Bm*HAT antigen (Table 2). Culture supernatants of PBMC from macaque #4996 and #5254 only had background levels of IFN-γ similar to that of the PBMC from control macaques (Table 2).

**Table 2:** Increase in IFN- γ production following r*Bm*HAT antigen stimulation.

	Control (alum only)					r*Bm*HAT(Immunized with r*Bm*HAT + alum)				
**Animal I.D**	4995	5240	5249	5252	5253	4996	5242	5254	5258	5259
**IFN-γ secretion (pg/ml)**	0	0	0	0	0	0	62.5	0	62.5	62.5

PBMC were isolated after the final immunization and subjected to r*Bm*HAT antigen stimulation. Supernatants from PBMC cultures were harvested upon 5 days of stimulation and assayed in ELISA for IFN- production.

### Anti-r*Bm*HAT antibodies in the sera of immunized macaques can participate in the killing of *B. malayi* L3

To determine the protective ability of anti-r*Bm*HAT antibodies in the sera of immunized macaques, we performed an *in vitro* ADCC assay. Results showed that the PBMC from vaccinated macaque were able to participate in the killing of 35% of *B. malayi* L3. When sera from individual macaques were evaluated maximum killing potential in the ADCC was 45% in the sera of macaque #5258 (Table 3). Sera from macaque #5242 and #5259 also showed significant killing potential with 38% and 35% killing respectively. Sera from macaque #4996 and #5259 had the least ADCC property with 25% and 31% killing respectively (Table 3). No larval death occurred when sera from control macaques were used in these assays (Table 3).

**Table 3:** Sera from macaques immunized with r*Bm*HAT participated in the killing of *B. malayi* L3 in an ADCC mechanism.

Animal I.D	Live L3 Mean ± S.Dof five wells	Dead L3 Mean ± S.Dof five wells	% Larval death Mean ± S.Dof five wells	Mean %Larval death
4995	10	0	0	0 (Control)
5240	10	0	0	
5249	10	0	0	
5252	10	0	0	
5253	10	0	0	
4996	7.5±0.5735	1.5±0.5735	25±5.16931*	35% ±6.123724* (Immunized)
5242	6.5±0.57735	4±0.57735	38±6.914*	
5254	6.5±1	3±0.5735	31±7.3981*	
5258	6.5±1.5275	5±0.57735	45±6.265021*	
5259	7±1.154701	3.5±1.154701	35±11.54701*	

50 µl of sera samples from r*Bm*HAT immunized macaques or control macaques were incubated with 2×10^5^ PBMC from naive macaques and 10 *B. malayi* L3 for 48 hrs at 37°C. Larval viability was evaluated at the end of the incubation.

Significant larval death **(P<0.05) compare to other macaques. Control wells were L3 incubated with media, cells alone or sera alone.

### Immunization with r*Bm*HAT conferred partial protection in macaques

**Figure 4:**
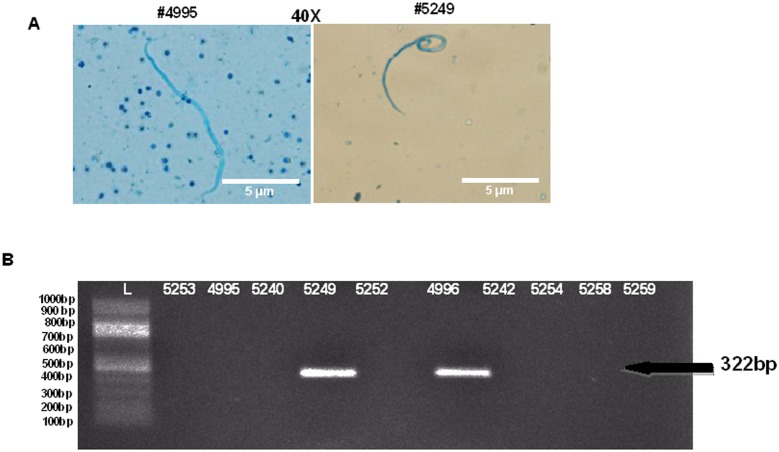
All 10 monkeys challenged with *B. malayi* L3 were screened for the appearance of Mf in the peripheral blood circulation by Knott technique and PCR analysis. A) Results from Knott technique showed the presence of mf in the blood that was stained with methylene blue. B) Mf specific *Hha I* gene was amplified and analyzed in 1% agarose gel.

One month after the final vaccination, all 10 monkeys were challenged with 500 *B. malayi* L3 and screened for the appearance of Mf in the peripheral blood circulation. A Knott test and PCR analysis was used to detect Mf. The Knott test was performed weekly from week 5 post challenge until the animals became positive. In our studies, challenged macaques became positive for Mf starting from week 10 post challenge. During weeks 11–20 post challenge, 3 of the control macaques became positive for Mf. Unfortunately, the rest 2 control macaques remained negative through the end of the study. In the vaccinated group, 3 of the macaques (#5242, #5254 and #5259) remained negative throughout the study. However, 2 of the vaccinated macaques (#4996 and #5258) became positive for Mf (Table 4 and Fig. 4A). To further confirm the infection, we performed a PCR analysis, where *Hha1* antigen specific primers were used to amplify for the presence of Mf specific DNA in the blood of infected monkeys. PCR analysis confirmed infections in macaque #5249 and #4996 (Table 4 and Fig. 4B). The other three positive animals identified by Knott technique were negative by PCR.

**Table 4:** Detection of Mf in the macaques challenged with *B. malayi* L3.

Macaque groups	Animal ID	Presence of Mf	
		Knott test	PCR test
Control (Immunized with only alum)	4995	+	−
	5240	−	−
	5249	+	+
	5252	−	−
	5253	+	−
r*Bm*HAT (Immunized with r*Bm*HAT mixed with alum)	4996	+	+
	5242	−	−
	5254	+	−
	5258	−	−
	5259	−	−

The appearance of Mf in the peripheral blood of macaques during weeks 11–20 post challenge was detected by Knott and PCR techniques. 3 macaques in control group and 2 macaques in the immunized group were found to be microfilaremic.

### r*Bm*HAT responding cells were present in the PBMC of immunized rhesus macaques after challenge

PBMC collected 10 weeks post challenge was stimulated with r*Bm*HAT to determine the antigen specific T cell response. PBMC of 3 animals #5242 (S.I- 0.928±0.01), #5258 (S.I- 1.091±0.16) and #5256 (S.I.-1.0181±0.13) from the vaccinated group that were negative for Mf showed significant proliferation upon r*Bm*HAT stimulation. Whereas, 2 of the vaccinated animals #4996 (S.I- 0.258±0.12) and #5254 (S.I- 0.379±0.03) positive for Mf did not show significant proliferation upon r*Bm*HAT stimulation (Table-5). No significant proliferation was observed in any of the control animals #4995 (S.I-0.280±0.03), 5240 (S.I. - 0.415±0.09), 5249 (S.I. - 0.300±0.26), 5252 (S.I. - 0.507±0.03) or 5253 (S.I - 0.475±0.25). S.I of PBMC stimulated with Concanavalin was in the range of 2.0–3.8.

### Eosinophil numbers were high in infected macaques showing Mf

**Figure 5:**
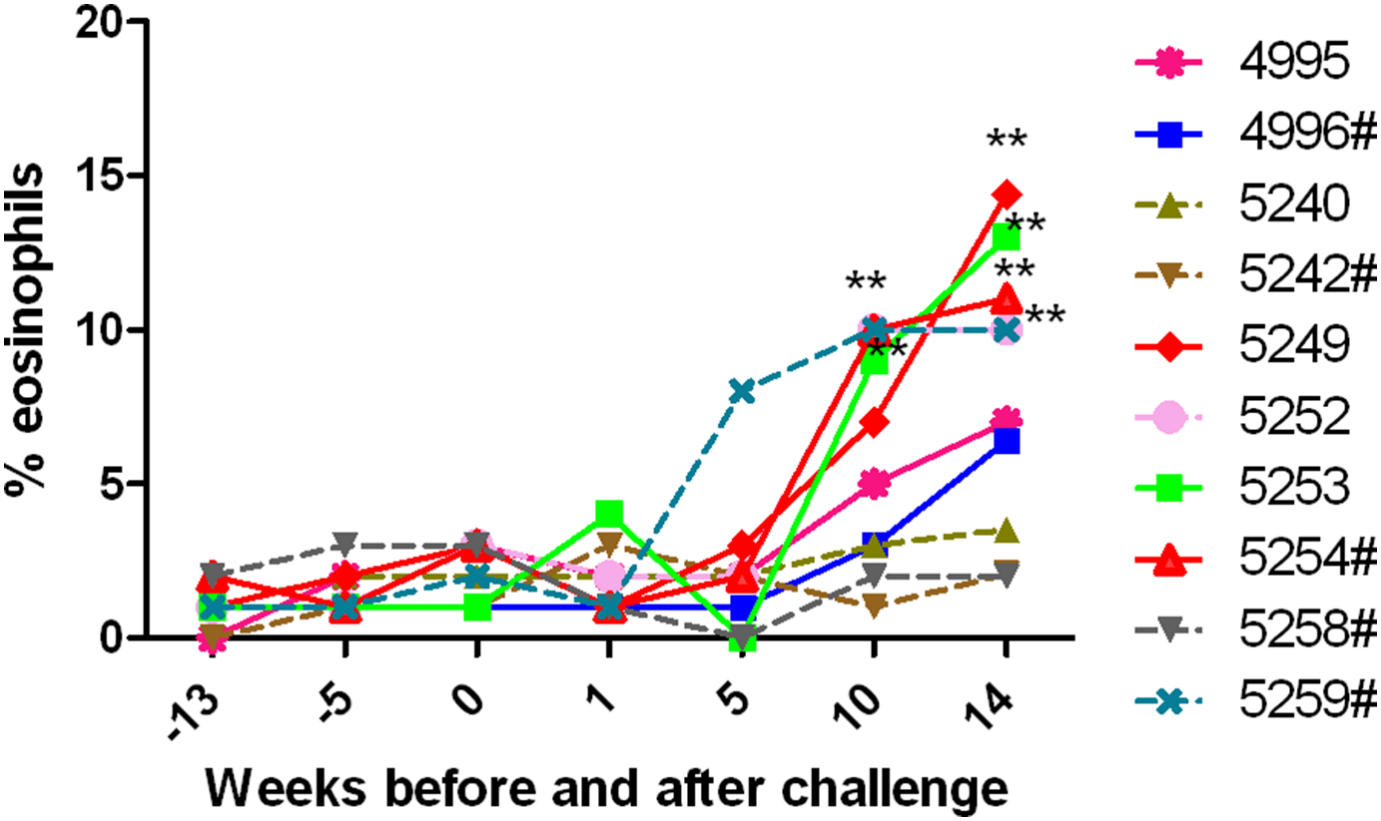
Percent of eosinophils in the peripheral blood of control and vaccinated macaques. Percent of eosinophils were evaluated in the peripheral blood of macaque pre- and post- challenge. Following challenge there was an increase in the frequency of eosinophils in animals that were microfilaremic as determined by Knott’s technique. Each line represents the eosinophil count of individual macaque on weeks −13, −9, −5, −1 before challenge, on day of challenge (week 0) and at weeks 1, 5, 10, and 14 post challenges. The levels of eosinophils in Mf positive animals are represented by a solid line. Levels of eosinophils in Mf negative animals are represented as a dotted line. # Macaques immunized with r*Bm*HAT. Significant at **P<0.001 high number of eosinophils.

Microfilaremic individuals show high eosinophil counts in their blood [23], [24]. A similar finding was observed in rhesus macaques as well. Absolute counts of eosinophils were determined on weeks, 13, 9, and 5 prior to challenge, on the day of challenge and on weeks 1, 5, 10, and 14 post challenge. Our results show that there was an increase in the frequency of eosinophil numbers in the peripheral blood of microfilaremic macaques around 10 weeks post challenges (Fig. 5). One macaque (#5259) that was negative for Mf also showed some eosinophilia. Eosinophil counts were 10 fold higher in control macaques that had microfilariae in their peripheral blood.

### High titer of antigen-specific IgG antibodies and elevated antigen specific secretion of IFN-γ from PBMC correlated with protection in the immunized macaques

**Figure 6:**
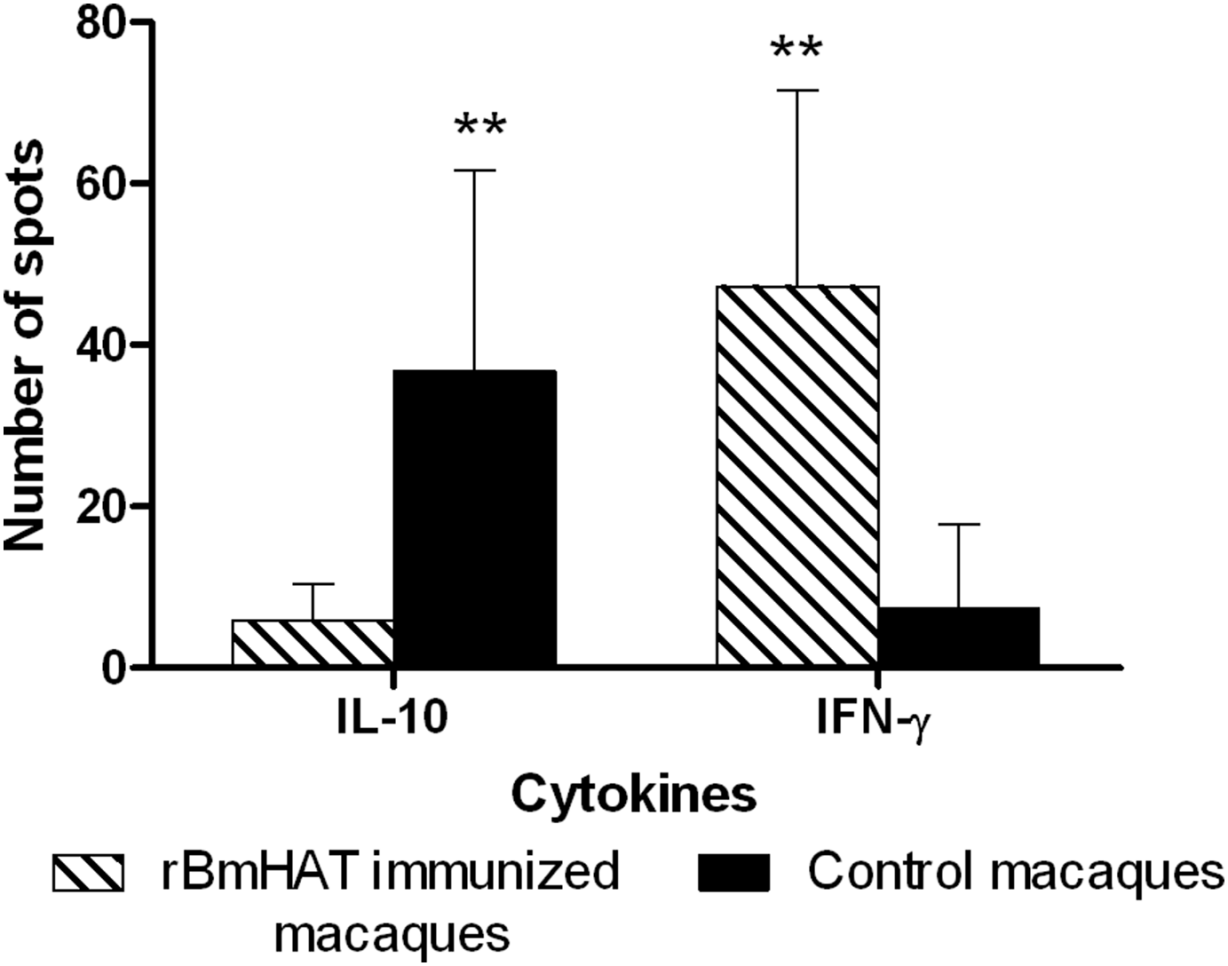
Antigen specific IFN-γ and IL-10 secreting cells in macaques. To determine the antigen-specific IFN-γ and IL-10 secreting cells ELISPOT was performed in the PBMC collected 20 weeks post challenge from vaccinated and control macaques. 1×10^6 ^cells/ml were stimulated with 100 ng/well of *B. malayi* adult soluble antigen (*Bm*A) for 48 hrs at 5% CO_2_ and used in the ELISPOT assays. Spot forming units were counted using a dissecting microscope. Antigen-specific cells were counted by subtracting the number of spots in the negative control wells from the wells containing antigens. Results are shown as the mean spot forming units obtained from triplicate wells. Each bar represents mean ± S.D of 5 animals. Significant **P<0.001 number of spots compared to other groups.

Since two of the macaques in the immunized group showed presence of infection following challenge, we compared the vaccine induced immune responses in the 2 infected macaques with similar responses in the 3 uninfected macaques within the immunized group. We compared the values before and after challenge. Values before challenge will eliminate any bias due to the challenge of parasites. A chart showing comparative immunological values are presented in Table 5. Results show that the titer of IgG antibodies was significantly high in the 3 immunized macaques that did not develop the infection after the challenge. Similarly PBMC from the same 3 macaques secreted higher levels of IFN-γ when stimulated with the r*Bm*HAT antigen. PBMC from the 2 immunized macaques that developed the infection after challenge were unable to secrete similar levels of IFN-γ in response to r*Bm*HAT stimulation. An ELISPOT assay was performed using PBMC from vaccinated and control macaques. Results show that in all the infected macaques there was a significant increase in the number of antigen-specific IL-10 secreting cells compared to IFN-γ secreting cells (Fig. 6). When we compared the ratio of IFN-γ to IL-10 secreting cells in the PBMC of immunized macaques, there was a significant increase in the IL-10 secreting cells in the two vaccinated macaques that showed infection (Table 6). These findings suggest a clear correlation between the type immune responses elicited and the failure to establish infection in the vaccinated macaques.

**Table 5:** PBMC were isolated 10 weeks post challenge from each macaque and cultured at 2×10^5^ cells per well in triplicate wells of a 96 wells plate.

Macaque groups	Animal ID	PBMC Proliferation Mean S.I ± S.D (N = 3)	
		Stimulated withConcanavalin A	Stimulated with r*Bm*HAT
Control (Immunized with only alum)	4995	3.260±0.01	0.280±0.03
	5240	3.090±0.58	0.415±0.09
	5249	2.982±0.24	0.300±0.26
	5252	3.674±0.83	0.507±0.03
	5253	2.582±0.72	0.475±0.25
r*Bm*HAT (Immunized with r*Bm*HAT mixed with alum)	4996	3.874±0.47	0.258±0.12
	5242	2.170±0.43	0.928±0.01**
	5254	2.068±0.18	0.379±0.03
	5258	3.304±0.64	1.091±0.16**
	5259	2.883±0.27	1.0181±0.13**

PBMC were stimulated *in vitro* with 1 µg/ml of r*Bm*HAT for 72 hrs at 37°C and analyzed for proliferation using cell counting kit (CCK-8). Con A was used as positive control. Stimulation index (S.I.) was calculated from the unstimulated control wells.

Significant proliferation of PBMC **(P<0.001) compare to PBMC from other macaques.

**Table 6:** Correlates of protection in macaques vaccinated with r*Bm*HAT.

Macaque groups	Animal ID	Immunological values before L3 challenge				Immunological values after L3 challenge	
		Antibody titer of>12,000 againstr*Bm*HSP	Antibody titerof >12,000 againstr*Bm*ALT-2	Antibody titer of>12,000 againstr*Bm*TSP LEL	IFN-γproduction	Mfdetection	Ratio of IFNγ:IL-10 secreting cells
Control (Immunizedwith only alum)	**4995**	−	−	−	−	+	1∶3
	**5240**	−	−	−	−	−	1∶1
	**5249**	−	−	−	−	+	1∶11
	**5252**	−	−	−	−	−	1∶0.01
	**5253**	−	−	−	−	+	1∶13
r*Bm*HAT (Immunized withr*Bm*HAT mixed with alum)	**4996**	−	−	+	−	+	1∶4
	**5242**	+	+	+	+	−	1∶0.003
	**5254**	−	−	+	−	+	1∶2
	**5258**	+	+	+	+	−	1∶0.001
	**5259**	+	+	−	+	−	1∶0.02

The three vaccinated macaques that did not show any evidence of infection had high titer of anti- r*Bm*HAT IgG antibodies and their PBMC secreted significantly high levels of IFN-γ but low levels of IL-10 as determined by an ELISPOT assay. Ratio of IFN-γ: IL-10 secreting cells was calculated for each macaque. These analyses also confirmed that there is a clear difference between the infected macaques and the three vaccinated macaques that did not show any infection. These findings coupled with the ADCC data clearly suggest that the three vaccinated macaques that did not show any signs of infection were protected.

#PBMC collected before challenge and stimulated with r*Bm*HAT.

## Discussion

Filarial parasites have complex life cycle that involve several discrete developmental stages within the human host [25]. L3 transmitted by mosquitoes develop into adult parasites that live in the lymphatic system for several years often more than 30 years evading the host immune system. Microfilariae that are released from these adult parasites that circulate in the blood can be cleared by anti-filarial drugs such as DEC, Albendazole and Ivermectin [26]–[28]. Current mass drug treatment administration (MDA) strategy uses these drugs to clear the parasite from the circulation to reduce the incidence of the infection and thus leading the way for elimination of lymphatic filariasis from the endemic regions. However, for sustained control strategy and for eradication of lymphatic filariasis, there is a need for an effective vaccine that can be combined with the current successful MDA approach. Previous studies from our laboratory showed that a multivalent fusion protein vaccine r*Bm*HAT, could confer over 95% protection against challenge infections in rodents. In this study we show that r*Bm*HAT vaccine confers approximately 35% protection in vaccinated macaques, as confirmed by the absence of circulating microfilaria, presence of protective antibodies and cells, absence of circulating antigen and lack of any visible pathology following a challenge infection. Our results showed that the protection in vaccinated macaques correlated with high titer of IgG antibodies against the vaccine antigen and higher frequency of IFN-γ secreting antigen responding cells in the peripheral blood and significant L3 killing potential in the sera of immune macaques. These findings suggested that the r*Bm*HAT vaccine is partially protective in macaques.

None of the vaccinated animals showed any adverse reactions to r*Bm*HAT vaccination. There were no abnormal clinical signs such as increase in the body temperature, pain, irritability or any altered behavioral changes in vaccinated macaques. Similarly the social behavior, feeding and sleeping habits were not altered in vaccinated macaques suggesting that the vaccination was safe in these macaques.

All the vaccinated macaques developed significantly high titers of IgG antibodies against r*Bm*HAT. This suggests that the vaccine antigens are highly immunogenic in macaques. These findings were similar to that observed in mice and gerbils following vaccination with r*Bm*HAT [9], [29]. Subsequent analysis of the isotype of IgG antibodies showed that anti-*Bm*HAT antibodies in the sera of vaccinated macaques were predominantly of IgG1 isotype. Surprisingly the vaccinated macaques did not show any IgG2 or IgG3 antibodies against the vaccine antigen. Previous studies in EN human subjects showed that these individuals carry significantly high titers of IgG1, IgG2 and IgG3 antibodies against the three vaccine antigens in their sera and these antibodies have protective function [3], [9]. Depletion of total IgG, IgG1, IgG2 or IgG3 antibodies from the EN sera significantly reduced the L3 killing ability of EN sera. Reconstitution of the antibody depleted sera with the antibodies recovered the L3 killing effect suggesting that the IgG1, IgG2 and IgG3 antibodies have an important role in the protection against lymphatic filariasis in the human [9]. Lack of elicitation of anti-r*Bm*HAT IgG2 and IgG3 antibodies in the sera of vaccinated macaques might explain the low protection rate observed following the challenge infections. Thus, it is possible that generating a balanced IgG1/IgG2 response in the macaque might significantly improve the protection rate after vaccination. Typically macaques do not produce IgG3 antibodies [30]. This might explain why we did not see any detectable anti-r*Bm*HAT IgG3 antibodies in our vaccinated macaques. Similarly, there were no detectable levels of anti-r*Bm*HAT IgE antibodies in the sera of vaccinated macaques. This is really advantageous if this vaccine were to be developed further for human use.

Alum adjuvants in general promote IgG1 antibodies and are poor inducers of IgG2 antibody responses [31]. Thus, the high levels of anti-r*Bm*HAT IgG1 antibodies observed in our vaccinated macaques may be largely alum driven. Therefore, we hypothesize that addition of another adjuvant such as Toll-like receptor (TLR)-4 agonists along with alum can promote both IgG1 and IgG2 responses. In fact, one of our recent studies in mice showed that a combination of alum plus TLR-4 agonist can induce a balanced Th1/Th2 response in mice and this correlated with significant protection [32]. Based on these studies, it is possible that an inclusion of TLR-4 agonist along with alum as adjuvants for r*Bm*HAT vaccination in macaques could substantially increase the protection rate.

Previous studies from our laboratory and others showed that ADCC assay could be used as a surrogate to measure protective responses to lymphatic filariasis *in vitro* [3], [9]. Our results showed that sera samples from all vaccinated macaque were able to kill *B. malayi* L3. However, the mean larval killing potential of the sera was only 35%, which was significantly lower than what we observed in the putatively immune EN human subjects and in the rodent models. Since both IgG1 and IgG2 antibodies appears to be critical for the killing of L3, we believe that the reduced ADCC function observed in the sera of our vaccinated macaque may be due to the lack of IgG2 antibodies.

Acute lymphatic filariasis infection is confirmed in the infected human subjects by demonstrating the presence of microfilariae in the peripheral blood using a Knott technique [33]. A similar analysis in our infected control macaques showed that microfilariae could be demonstrated in the peripheral blood of infected macaques. In addition we also used a PCR based method to confirm the infection [21]. PCR method detects the circulating antigens in the peripheral blood of infected animals. Both these analyses are mutually exclusive but provided a confirmative diagnosis. However, the PCR approach in our hands appeared to be less sensitive than actual demonstration of the parasite. Out of the three animals that were positive for microfilariae by Knott technique in the control group only one animal was found to be positive by the PCR method. Similarly out of the 2 positive samples identified by Knott’s method in the vaccinated animals, only one animal was positive in the PCR assay. It is not clear why we observed this discrepancy. One possible explanation may be that we identified one Mf in 50 ml of blood by the Knott’s technique and the PCR method failed to detect low levels of infections. These findings suggest that multiple diagnostic approaches may be needed to confirm the presence of low level infections. Nevertheless, samples positive by PCR method was also positive by Knott’s technique. Demonstrating Mf in the peripheral blood inconclusively proves infection.

Two of the control macaques in our study did not pick the infection. At this time we do not know why the infection failed to establish in these two macaques. Prior to entering the animals into the study, serological and parasitological analyses were performed in all the animals and all of them were negative for any parasitic infection and did not carry any antibodies against *B. malayi*.

A distinctive characteristic of lymphatic filariasis infection in the human is the appearance of eosinophilia [24]. All the infected macaques in the control and vaccinated groups showed significant eosinophilia, whereas, eosinophilia was not a prominent finding in uninfected animals. One vaccinated animal with no infection also showed eosinophilia. Clinical data did not show any signs of other infections or allergy in this particular animal. Reason for elevated eosinophils in this animal could not be determined.

PBMC hypo-responsiveness to parasite antigen is another characteristic in human subjects who have circulating microfilaria [34], [35]. Analysis of the proliferative responses of PBMC from the 2 infected macaques in the vaccinated group with *Bm*A and r*Bm*HAT antigens showed minimal proliferation similar to the control animals further confirming that these animals were infected, whereas, the PBMC from all the three uninfected animals in the vaccinated group proliferated significantly suggesting that these animals are immune.

PBMC from lymphatic filariasis infected patients do not secrete interferon (IFN)-γ in response to parasite antigen [35]–[37] but secrete significant amounts of the anti-inflammatory cytokines primarily IL-10 because of circulating microfilariae [38]. Analysis of the cytokine response of PBMC from the two infected macaques in the vaccinated group showed that these cells secrete high levels of IL-10 and low levels of IFN-γ compared to PBMC from the three uninfected macaques which secreted significantly high levels of IFN-γ. A similar dichotomy in the IFN-γ secretion was seen in the mouse model as well [9]. These findings clearly showed that the three Mf negative macaques in the vaccinated group have developed significant immunity against the challenge infection and prevented establishment of the infection.

Thus, based on high titer of antibody responses elicited after vaccination in macaques, ability of the serum antibodies to participate in the ADCC function to kill *B. malayi* L3, absence of Mf in the peripheral circulation, significant proliferative response of PBMC and their ability to secrete significant amounts of IFN-γ it could be concluded that the three Mf negative macaques in the vaccinated group are truly protected. Unfortunately, two of the macaques in the control group failed to show any infection. Analysis of the various parameters in these two uninfected control macaques definitely demonstrated that the challenge infection failed to establish in these two macaques. Therefore, we conclude that vaccination with r*Bm*HAT can confer partial protection in macaques against *B. malayi* L3 challenge infection. Further studies are needed to improve the vaccine formulation or adjuvant formulation to increase the vaccine induced protection against *B. malayi* infections in macaques before moving this vaccine to human clinical trials.
